# Global gene expression analysis in time series following N-acetyl L-cysteine induced epithelial differentiation of human normal and cancer cells *in vitro*

**DOI:** 10.1186/1471-2407-5-75

**Published:** 2005-07-07

**Authors:** Anna C Gustafsson, Ilya Kupershmidt, Esther Edlundh-Rose, Giulia Greco, Annalucia Serafino, Eva K Krasnowska, Thomas Lundeberg, Luisa Bracci-Laudiero, Maria-Concetta Romano, Tiziana Parasassi, Joakim Lundeberg

**Affiliations:** 1 Royal Institute of Technology, AlbaNova University Center, Department of Biotechnology, Roslagstullsbacken 21, SE-106 91 Stockholm, Sweden; 2 Silicon Genetics, 2601 Spring Street, Redwood City, California 94063, USA; 3 Istituto di Neurobiologia e Medicina Molecolare, CNR, Viale Marx 15-43, 00137 Roma, Italy; 4 Rehabilitation Medicine, Karolinska University Hospital, 117 76 Stockholm, Sweden; 5 Associazione Italiana Iniziativa Medicina Sociale, Corso Trieste 16, 00185 Roma, Italy

## Abstract

**Background:**

Cancer prevention trials using different types of antioxidant supplements have been carried out at several occasions and one of the investigated compounds has been the antioxidant N-acetyl-L-cysteine (NAC). Studies at the cellular level have previously demonstrated that a single supplementation of NAC induces a ten-fold more rapid differentiation in normal primary human keratinocytes as well as a reversion of a colon carcinoma cell line from neoplastic proliferation to apical-basolateral differentiation [1]. The investigated cells showed an early change in the organization of the cytoskeleton, several newly established adherens junctions with E-cadherin/β-catenin complexes and increased focal adhesions, all features characterizing the differentiation process.

**Methods:**

In order to investigate the molecular mechanisms underlying the proliferation arrest and accelerated differentiation induced by NAC treatment of NHEK and Caco-2 cells *in vitro*, we performed global gene expression analysis of NAC treated cells in a time series (1, 12 and 24 hours post NAC treatment) using the Affymetrix GeneChip™ Human Genome U95Av2 chip, which contains approximately 12,000 previously characterized sequences. The treated samples were compared to the corresponding untreated culture at the same time point.

**Results:**

Microarray data analysis revealed an increasing number of differentially expressed transcripts over time upon NAC treatment. The early response (1 hour) was transient, while a constitutive trend was commonly found among genes differentially regulated at later time points (12 and 24 hours). Connections to the induction of differentiation and inhibition of growth were identified for a majority of up- and down-regulated genes. All of the observed transcriptional changes, except for seven genes, were unique to either cell line. Only one gene, *ID-1*, was mutually regulated at 1 hour post treatment and might represent a common mediator of early NAC action.

The detection of several genes that previously have been identified as stimulated or repressed during the differentiation of NHEK and Caco-2 provided validation of results. In addition, real-time kinetic PCR analysis of selected genes also verified the differential regulation as identified by the microarray platform.

**Conclusion:**

NAC induces a limited and transient early response followed by a more consistent and extensively different expression at later time points in both the normal and cancer cell lines investigated. The responses are largely related to inhibition of proliferation and stimulation of differentiation in both cell types but are almost completely lineage specific. *ID-1 *is indicated as an early mediator of NAC action.

## Background

Most human cancers arise in epithelial cells, underlining the importance of understanding the molecular biology of cancer and the complex balance of proliferation and differentiation in this cell type. Increased knowledge of these processes may provide unique targets for the future development of pharmacotherapy aiming at halting or reversing metastasis and cancer growth.

N-acetyl-L-cysteine (NAC) is a membrane permeable aminothiol that functions as a nucleophilic ROS scavenger and antioxidant as well as a precursor of intracellular cysteine and glutathione (GSH). The reduced cysteine represents the active form, as opposed to the inactive oxidized cystine dimer. To date, NAC is used as a mucolytic and as acute treatment of fulminant hepatic failure following paracetamol poisoning. However, cancer preventing and therapeutic effects have also been suggested. In particular, NAC has been demonstrated to induce anti-proliferative and differentiating effects in normal human epidermal keratinocytes (NHEK), as well as in the epithelial colon cancer cell line Caco-2 [1]. Primary normal human epidermal keratinocytes (NHEK) undergo spontaneous terminal differentiation over 30 days in culture. However, if supplemented with 2 mM NAC 24 hrs after seeding, an accelerated differentiation process can be observed. Three days post NAC exposure, differentiation of NHEK is demonstrated by an increased number of intercellular junctions, basal localization of cytokeratin and apical localization of actin determined by scanning electron micrographs of cells and sub-structures and high resolution confocal fluorescence immuno micrographs of for example β-catenin, E-cadherin, actin and cytokeratins. Furthermore ceased proliferation can be demonstrated by ^3^H thymidine incorporation without accompanying apoptosis experimentally verified by properdium iodide labelling and flow cytometry. Interestingly, an epithelial colon cancer cell line responded in analogy with the normal epithelial cells. Caco-2 differentiate spontaneously over a period of around 25–30 days in culture [2]. However, when a single supplement of 10 mM NAC was given to Caco-2 cells 24 hrs after seeding, the proliferation decreased and the cells progressed to a differentiated state in three days without any sign of apoptosis [1]. Here the differences in gene expression was studied overtime for both NHEK and Caco-2 cells using microarray technology with subsequent confirmation of a selected set of genes. The results are discussed from the perspective of accelerated differentiation and growth arrest.

## Methods

### Cell cultures

Normal human epidermal keratinocytes, NHEK (Cambrex, San Diego, CA), plated at a density of 8 × 10^3 ^cells/cm^2^, were grown in KGM^® ^medium plus KGM^® ^SingleQuots^® ^(Cambrex). The Caco-2 human colon carcinoma cells were seeded at a density of 9 × 10^3 ^cells/cm^2^, grown in Dulbecco's modified Eagle minimum essential medium (DMEM, GIBCO Labs, Grand Island, N.Y.), supplemented with 10% (v/v) heat-inactivated fetal calf serum (GIBCO Labs), L-glutamine (2 mM), penicillin (50 IU/ml) and streptomycin (50 mg/ml). The N-acetyl-L-cysteine (NAC, Sigma Chem. Co., St Louis, MO) stock solution (20 mM in KGM and 100 mM in DMEM) was stored at 4°C in the dark and used within 1 week from preparation. Twenty-four hrs after seeding, filter sterilized NAC solution was added to the cell cultures to a final concentration of 2 mM and 10 mM in NHEK and Caco-2 respectively. The concentrations were selected after performing a dose-dependent inhibition analysis for each of the two cell types [1].

### Scanning electron microscopy

Cells grown on coverslips were fixed with 2.5% glutharaldehyde in 0.1 M Millonig's phosphate buffer (MPB) at 4°C for 1 hr. After washing in MPB, cells were post-fixed with 1% OsO_4 _in the same buffer for 1 hr at 4°C and dehydrated in increasing acetone concentrations. The specimens were critical-point dried using liquid CO_2 _and sputter-coated with gold before examination on a Stereoscan 240 scanning electron microscope (Cambridge Instr., Cambridge, UK).

### Experimental set up

From the normal (NHEK) cells, RNA was obtained from cultures grown for 1, 12 and 24 hrs after NAC treatment as well as from untreated cells at the same time points. Replicates at the level of individually performed cDNA synthesis were used for hybridisation in duplicate for the 1, 12 and 24 h time points except for one fall out of a 24 hrs post NAC treated sample. RNA was extracted from both treated and non-treated cancer (Caco-2) cell cultures at 1, 12 and 24 hrs. Biological replicates were used for hybridisation in a duplicate fashion for both treated and non-treated samples.

### RNA extraction, purification and quality control

The total RNA was extracted from cell cultures using Trizol (Gibco BRL, NY, USA) according to the manufacturers instructions. Thereafter mRNA was extracted by oligo dT Dynabeads (Dynal, Oslo, Norway) and the quality of mRNA was validated using the Bioanalyzer 2100 (Agilent technologies, Waldbrunn, Germany).

### Target preparation and hybridisation

A total of 8 micrograms of mRNA from each sample was used to perform cDNA synthesis. Following in vitro transcription, the biotin labelled cRNA was fragmented and a total of 15 micrograms were subsequently hybridised and analysed on the Affymetrix GeneChip™, all according to the manufacturers instructions (Affymetrix, Santa Clara, USA) with scanning performed on the GeneArray 2500A Scanner (Affymetrix, Santa Clara, USA).

### Data analysis

The MAS 5.0 software package (Affymetrix Inc.) was used to compute cell intensity files (.cel) for each chip. For the purpose of inspection of parameters important to quality control, chip files (.chp) were also generated for each chip using the statistical expression algorithm. Next, individual probeset intensity values were computed based on the cell intensity files using the RMA algorithm within the Bioconductor package. All data was analysed in three separate batches (2 separate sample preparation batches for the Caco-2 cell line and one for NHEK cell line. Each batch also included RNA samples obtained from 30 min, 3 hrs and 48 hrs treated and untreated cell cultures which are not presented in this manuscript). The RMA background correction, quantile normalization, only PM probe correction and a median polishing were applied. Next, data probeset intensities from each sample were converted to a biological fold ratio between the treated and control sample. To generate the ratio, signal intensities of each treated sample were divided by the control sample intensities for each corresponding time point, separately within each RMA batch. Because of a missing 24 hrs control for NHEK cell line due to fallout, time point 12 hrs control was used instead (detailed investigation showed that control samples at each time point were extremely similar to each other, rationalizing this choice). The average of replicates for each time point in two cell lines was used in the downstream analysis. Due to the quality control issues encountered with time point 24 hrs NHEK cell line, only one replicate was used. The standard deviation statistics was calculated using the Global Error Model based on deviation from one (GeneSpring, Silicon Genetics). A final total of 6 conditions were generated (1, 12, 24 hrs time points for NHEK and Caco-2). The MIAME compatible dataset is made available at the ArrayExpress expression data repository at EMBL.

In order to identify genes that show significant changes during treatment we searched for genes that show significant up- or down-regulation in treatment relative to the control, in at least one of the time point for one of the cell lines (filtering was done using the t-test p-value < 0.01, indicating significant deviation from the control value or from the ratio of 1). Two-way hierarchical clustering of genes and conditions was performed using the set of pre-filtered genes (2054) and Standard correlation. In order to identify genes with significant NAC treatment response at each time point for each cell line we applied a two-step filtering based on the t-test p-value of less than 0.1 (indicating statistically significant changes from the control value), and a biological fold cut-off of 1.5 fold (up or down). A total of 6 genelists (3 for each cell line) was produced and visualized graphically for common distinct patterns. Data filtering and visualization was performed using GeneSpring (Silicon Genetics). To assess the overrepresentation of functional groups, according to Gene Ontology, the publicly available tool EASE (v2.0) was used [3].

### Real-time kinetic PCR

Validation of microarray results was performed by quantifying relative mRNA expression levels of several genes of interest. Two RT-PCR formats were used for the investigated genes. The first relied on primers and TaqMan probes obtained from Applied Biosystems and their Assay-on-demand system and the second approach was based on in-house designed primers and SYBR green detection. The assays used either the glyceraldehyde-3-phosphate dehydrogenase (*GAPDH*) or the transferrin receptor (*TFR*) as the endogenous internal reference gene.

For the first method, cDNA synthesis was performed using SuperScript™ III (Invitrogen) in a 20 μl reaction containing: 1x first strand buffer, 5 mM DDT, 40 U RNasin (Promega), 5 μg total RNA, 200 U SuperScript™ III, 250 mM oligo(dT)20 primer, 0,5 mM dNTP each dATP, dGTP, dCTP, dTTP. Primer, total RNA and nucleotides were heated to 65°C for 5 min and subsequently cooled on ice for 1 min. After addition of the other reagents the samples were incubated at 50°C for approximately 60 min. Finally, the reaction was inactivated by heating to 70°C for 15 min. Assays-on-Demand (Applied Biosystems), containing gene specific primers and probes labelled with 6-carboxyfluorescein (FAM) at the 5' end and with 6-carboxytetramethylrhodamine (TAMRA) at the 3' end for the following genes: *GAPDH *(Genbank nb. BC029618), *MMP9 *(J05070), *AKR1C3 *(D17793), *AQP3 *(N74607), *PLAT *(M15518), *HBP1 *(AF019214), *PTGS2 *(U04636), *ERBB3 *(M34309) and *PNRC1 *(U03105) (see Table 3), were used together with TaqMan Universal PCR Master Mix (Applied Biosystems) in the TaqMan real time PCR reaction as described by the manufacturer. 25 μl reactions were done in 96-well plates. Amplification and detection was carried out using the iCycler iQ Multicolor Real-Time PCR Detection System (Bio-Rad Laboratories, Hercules, CA, USA). All samples were run in triplicate.

**Table 3:** Relative gene expression levels measured by real time kinetic PCR in Caco-2 and NHEK cells after NAC treatment as compared to untreated cells.

**Caco-2**												
**Gene**	**Common**	**Genbank**	**1h**		**12h**		**24h**		**48h**			
			**RT-PCR**	**Affymetrix**	**RT-PCR**	**Affymetrix**	**RT-PCR**	**Affymetrix**	**RT-PCR**	**Affymetrix**		

matrix metalloproteinase 9*	MMP9	J05070	na	nd	no change	up	na	up	na	na		
aldo-keto reductase*	AKR1C3	D17793	na	nd	no change	up	up (2.8)	nd	na	na		
aquaporin 3*	AQP3	N74607	no change	down	up (6.8)	up	up (5.0)	up	na	na		
plasminogen activator, tissue*	PLAT	M15518	na	nd	no change	nd	no change	nd	na	na		
HMG-box transcription factor 1*	HBP1	AF019214	no change	nd	up (2.6)	up	up (2.1)	nd	na	na		
Cox-2*	PTGS2	U04636	no change	nd	no change	nd	down (2.2)	down	na	na		
v-erb-b2*	ERBB3	M34309	na	nd	up (3.7)	up	no change	nd	na	na		
proline-rich nuclear receptor coactivator 1*	PNRC1	U03105	na	nd	no change	up	up (2.4)	nd	na	na		
inhibitor of differentiation*	ID1	AA457158	down (3.8)	down	na	nd	na	nd	na	nd		
E-cadherin**	CDH1	Z13009	na	nd	no change	nd	up	nd	no change	na		
hsp27**	HSPB1	U90906	na	nd	up	nd	up	nd	no change	na		
p53**	TP53	U94788	na	nd	up	nd	no change	nd	no change	na		
N-myc downstream regulated gene 1**	NDRG1	D87953	na	nd	up	nd	up	up	up	na		
**NHEK**												
			**30 min**		**3h**		**12h**		**24h**		**48h**	
			
**Gene**	**Common**	**Genbank**	**RT-PCR**	**Affymetrix 1 h**	**RT-PCR**	**Affymetrix**	**RT-PCR**	**Affymetrix**	**RT-PCR**	**Affymetrix**	**RT-PCR**	**Affymetrix**

matrix metalloproteinase 9*	MMP9	J05070	no change	nd	no change	nd	na	up	na	up	up (11.1)	nd
aldo-keto reductase*	AKR1C3	D17793	na	nd	down (4.0)	nd	na	nd	na	nd	down (5.3)	nd
aquaporin 3*	AQP3	N74607	no change	nd	no change	nd	na	nd	na	up	up (2.8)	nd
plasminogen activator, tissue*	PLAT	M15518	no change	nd	no change	nd	na	nd	na	down	no change	nd
HMG-box transcription factor 1*	HBP1	AF019214	down (6.3)	nd	down (4.0)	nd	na	up	na	nd	no change	nd
Cox-2*	PTGS2	U04636	down (7.7)	nd	down (6.3)	nd	na	nd	na	down	down (8.3)	nd
v-erb-b2*	ERBB3	M34309	na	nd	down (2.4)	nd	na	up	na	nd	down (4.5)	nd
proline-rich nuclear receptor coactivator 1*	PNRC1	U03105	na	nd	down (5.3)	nd	na	up	na	nd	no change	nd
inhibitor of differentiation*	ID1	AA457158	down (8.3)	down	na	nd	na	nd	na	nd	na	nd
E-cadherin**	CDH1	Z13009	na	nd	na	nd	no change	nd	up	nd	up	nd
hsp27**	HSPB1	U90906	na	nd	na	nd	up	nd	up	nd	up	nd
p53**	TP53	U94788	na	nd	na	nd	no change	nd	up	nd	up	nd
N-myc downstream regulated gene 1**	NDRG1	D87953	na	nd	na	nd	no change	nd	no change	nd	no change	nd

Significant fold change: <0.5 for down regulated and >2.0 for up regulated genes, respectively. na = not analysed; nd = no data. * Taqman RT-PCR was done on these genes using Assays-on-demand (Applied Biosystems). GAPDH was used as a reference gene. **SYBR green RT-PCR was done on these genes using in house designed primers. Transferrin receptor was used as a reference gene.

For the second method, in-house designed gene-specific primers for six human transcripts (Transferrin receptor (*TFR*): NM_003234 (5'-TCCCTAGGAGGCCGTTTCC-3'and 5'-GCCTACCCATTCGTGGTGAT-3), *COX-2*: NM_000963 (5'-GCATTGGAAACATCGACAGTGT-3'and 5'-TGACGTCTTTTTACTTGAATTTCAACTTATAT-3); *NDRG1*: NM_006096 (5'-CCAGTGCGGCTGCCAG-3'and 5'-TTCCTATGAGAAAATCCACGGTG-3); *TP53*: NM_000546 (5'-CCTTGAGGGTGCCTGTTCC-3'and 5'-CCCTCTACCTAACCAGCTGCC-3');*CDH1*: NM_004360 ('5'-TGAAGACCTTTAATGGCTTCCC-3'and 5'-CACACTTACTCAGAACAAGTCACTGG-3'); *HSPB1*: NM_001540) (5'-AAAATCCGATGAGACTGCCG-3' and 5'-GCACAGCCAGTGGCGG-3') were designed using the Primer Express software (Applied Biosystems, Foster City, CA, USA). Real time RT-PCR analysis was performed in triplicate using ds cDNA synthesized from mRNA obtained from the investigated cells. All PCRs were performed at 60°C annealing temperature and the *TFR *gene was used as internal standard. A PCR mastermixture was prepared using the SYBRGreen PCR Core Reagents (Applied Biosystems, Foster City, USA) and aliquoted into microplate wells together with 1 μl template and 5 pmol of each primer for a final volume of 25 μl per reaction. The iCycler (Bio-Rad) was used for PCR and detection of fluorescent signals. Standard curves (C_T _versus log concentration) were generated for each primer pair using duplicate cDNA samples in a series of consecutive 5-fold dilutions. Efficiency calculations (E=(10^(-1/amplificationslope)-1) were performed to validate compatibility of investigated genes with the internal control. The compatibility of all pair-wise compared amplification efficiencies were confirmed with a maximal deviation of 10% (data not shown). The specificity of all individual amplification reactions was confirmed by meltcurve analysis (data not shown).

The relative mRNA expression levels of the target genes in each sample were calculated using the comparative C_T _method. The C_T _value was defined as the number of PCR cycles required for the fluorescence signal to exceed the threshold value. C_T _values were defined as the absolute value of the difference between the C_T _of the target RNA and the Ct of the housekeeping gene RNA for each sample. The level of significance was set to a 2-fold relative difference between samples, i.e. significant fold change 0.5 < 2^-ΔΔC^_T _> 2 for down- and up-regulated genes respectively.

## Results

### Experimental overview

**Figure 1:**
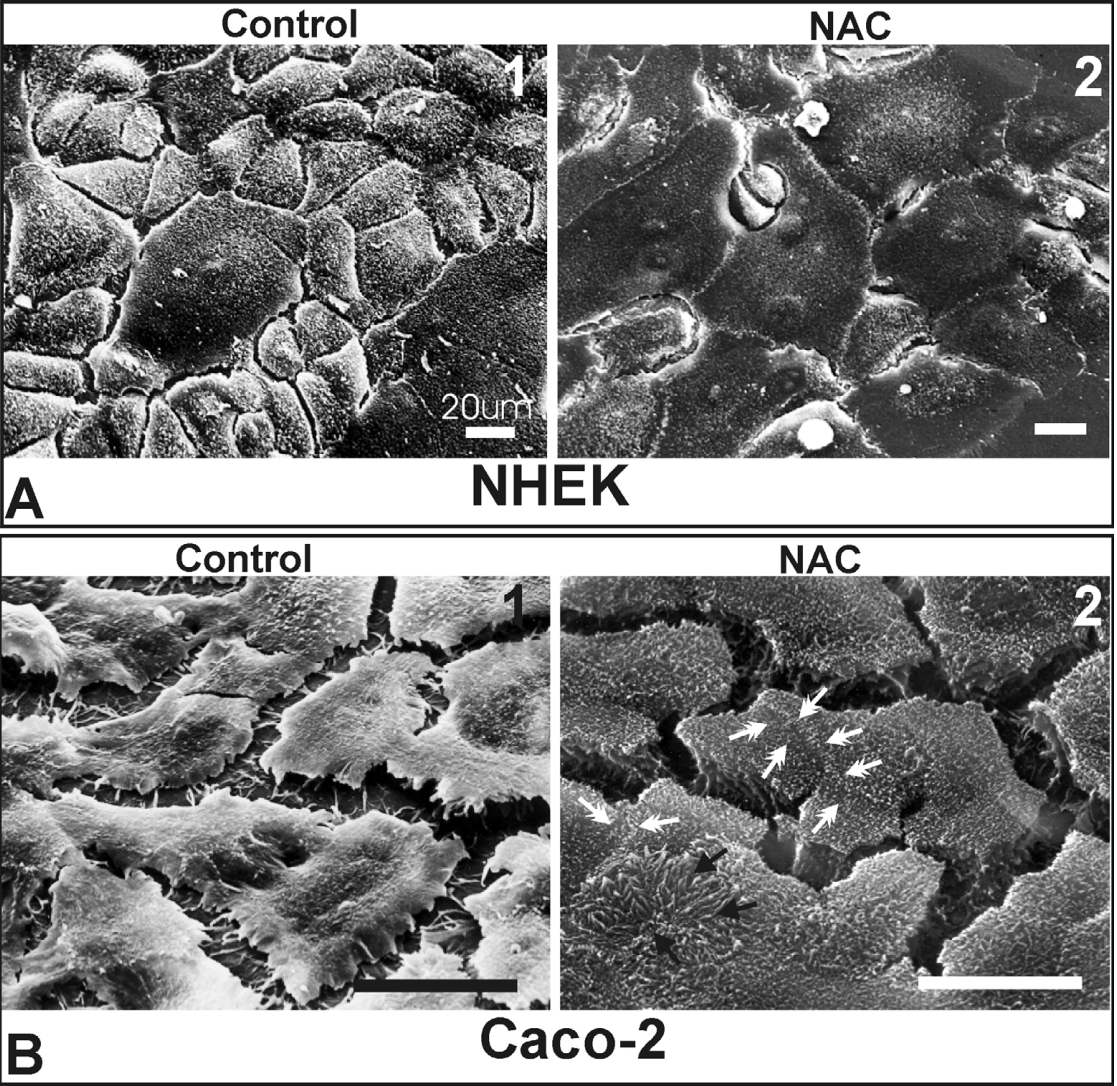
**Scanning electron micrographs showing the effect of NAC treatment on the morphology of NHEK (A) and Caco-2 (B) cells. **A1) NHEK untreated controls show a heterogeneous polygonal morphology, with a villous surface and relatively broad intercellular space; A2) After 72 h with 2 mM NAC cells grow flat in a thin monolayer with a regular polygonal morphology and a smooth surface with loss of the fine microvillous structure, also showing dramatically reduced intercellular space. B1) Proliferating Caco-2 cells display an irregular morphology with scarce microvillous structures and large intercellular space; B2) After 72 h with 2 mM NAC Caco-2 cells had the morphology of end-stage differentiated cells, with a regularly polygonal and about three times thicker than controls, with a relevant number of brush border microvilli at the cell surface (*black arrows*) as well as a dramatically reduced intercellular space (*white arrows*). *Bars*: 20 μm

The aim of this work was to study the molecular effects of the antioxidant N-actetyl- cysteine (NAC) on proliferating cells, by gene expression analysis using the Affymetrix GeneChip platform. Previous studies on cell culture systems, demonstrated mainly by morphological and biochemical data, have indicated that NAC addition stimulates proliferating cells to go into differentiation phase [1]. Figure 1 displays a scanning electro micrograph image of the cell types tested and demonstrates the morphological shift from proliferating to differentiating cells after addition of NAC. We have performed a microarray-based gene expression analysis of the human colon carcinoma cell line (Caco-2) and normal human epidermal keratinocytes (NHEK) over time (1, 12, and 24 hrs) after addition of NAC, compared to untreated, control samples at the same time points. Data obtained from GeneChip analysis were processed using the RMA analysis approach and samples were normalized to their corresponding controls. Filtering was done based on two criteria (p-value < 0.1 and >1.5 fold up-or down-regulation) for each time point. The labelling and hybridisation was done in duplicate, except at 24 hrs NAC treatment of NHEK, where a single measurement was made.

### Analysis

Gene lists of up- and down-regulated genes at each time point for NHEK and Caco-2 (1, 12, 24 hrs) were generated. In addition, time points within each cell type were analysed to identify similarities/differences among early and late time-point responses. Any results that produced zero genes were skipped. A summary of the top ten differentially expressed genes in Caco-2 and NHEK, at all time points, is depicted in additional files 1 and 2. The complete lists of differentially expressed genes are accessible as additional datafiles 3 and 4 or at .

There were no general differences in the number of transcripts detected in treated as compared to untreated samples in any of the cell lines. Following NAC induced differentiation in Caco-2 and NHEK, 253 and 414 targets, respectively, were significantly differentially expressed across the time series, according to above statistical criteria (Table 1). Generally, for both cell types, the amount of significantly up/down-regulated genes is limited to < 200 genes after consideration of multiple appearances. In Caco-2, a tendency towards more down-regulated genes was identified, while in NHEK, the numbers of genes induced was approximately equal to those repressed (Table 1).

**Table 1:** Number of genes induced/repressed at each time point.

**Time points**	**Caco-2**	**NHEK**
1 hr (repressed)	8	16
12 hrs (repressed)	54	10
24 hrs (repressed)	86	171
1 hrs (induced)	1	26
12 hrs (induced)	69	28
24 hrs (induced)	35	163

Both Caco-2 and NHEK exhibit a relatively limited early response at 1 hr, followed by an increasingly stronger response at 12 and 24 hrs. The initial response could be characterized as transient, since most of the genes induced/repressed initially either changed in the opposite direction later on, or simply went back to normal expression levels.

The induced/repressed genes in the two cell types are generally quite different despite their morphological similarities after NAC treatment. This is for example demonstrated in that most of the genes induced/repressed in Caco-2 show no change in expression levels in NHEK and vice versa (see below). A direct comparison of the corresponding differentially expressed genes is also provided.

### Caco-2 analysis

**Figure 2:**
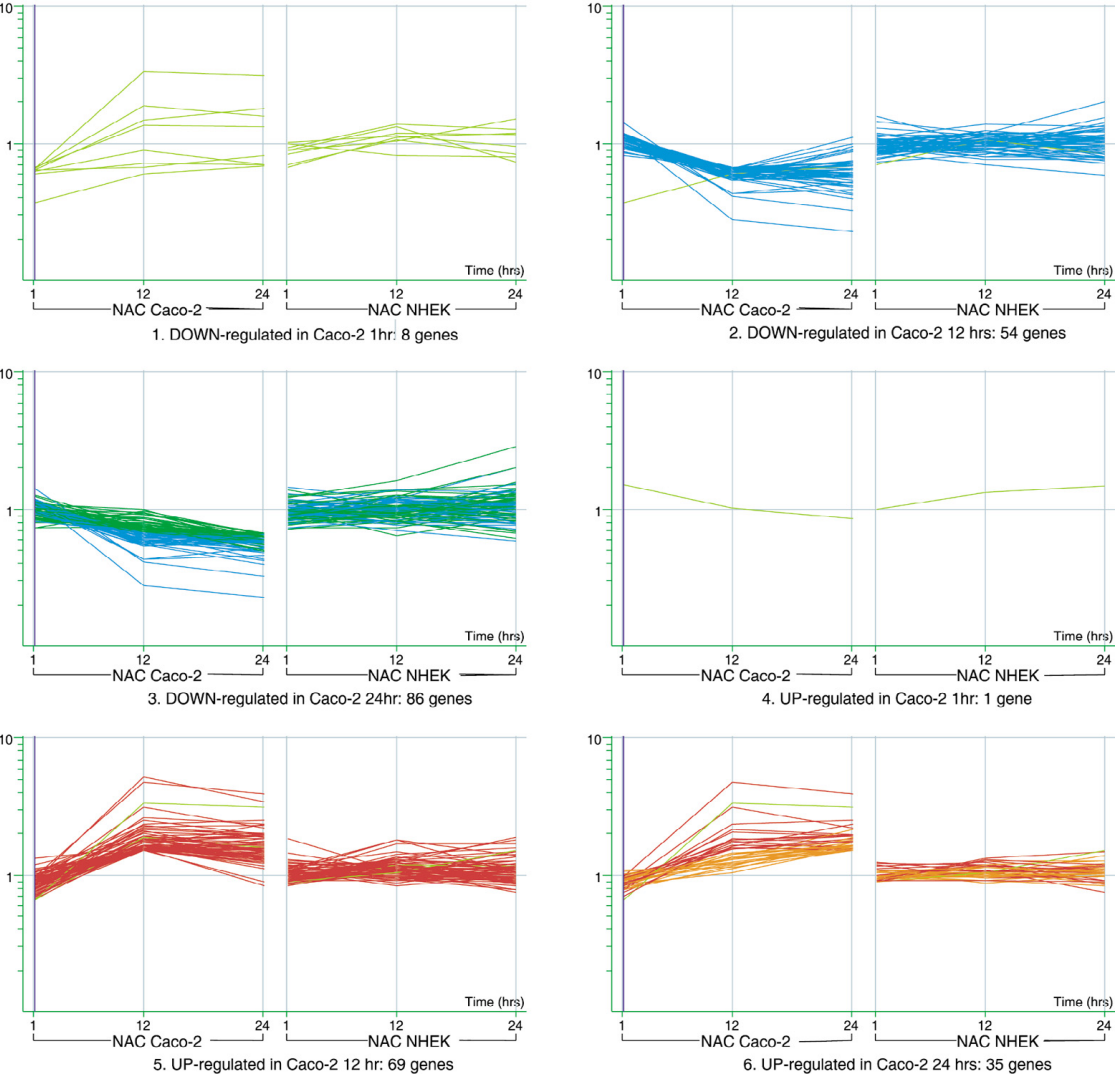
**Matrix view of significantly affected genes. **Genes induced/repressed in Caco-2 relative to control and their corresponding expression profiles in NHEK. The color is specific for genelist of affected genes at each time point (unless these are the same genes as from one of the earlier time points). Clearly, the early response at 1 hr is very limited (1,8 genes in Caco-2). There are also almost no genes induced/repressed at 1 hr that continue the trend at later time points – indicating an early transient response. This is markedly different for 12 hr and 24 hr time points that show many common genes. It is also clear that many genes affected by NAC treatment in one cell line don't show the same response in the other cell line.

The general trends for Caco-2 cells are depicted in Figure 2 based on the collection of genes that passed the set criteria.

The graph shows the behaviour of genes induced/repressed in Caco-2 across the time courses in both cell types (signal represents the fold difference between each treatment and the average of corresponding controls). As indicated above, there is a small transient change at 1 hr, and a more significant change, taking place at later time points. In particular, it is observed that the majority of genes repressed at 12 hrs continue their trend at 24 hrs (blue lines correspond to 12 hrs genes, dark green line corresponds to 24 hrs genes only). Similarly, many genes up-regulated at 12 hrs continue the trend at 24 hrs (red line 12 hrs genes, orange line 24 hrs genes only). (Gene lists showing multiple appearances in the same cell type, Caco-2, at more than one time point can be found in additional file 4 or at ). The expression pattern of the corresponding genes in the other cell system NHEK shows weak or no correlation.

Table 1 represents the number of genes induced/repressed at each time point for Caco-2 cells and NHEK cells, including those with changes across multiple time points. Again, it is evident that early activity (1 hr) is limited and quite different from the activity at later time points and that few genes induced/repressed early on (1 hr) continue their trend at later time points (Figure 2). This has also been observed after 24 and 48 hrs using another microarray platform based on spotted cDNA arrays (data not shown).

### NHEK analysis

The NHEK cells were analysed in a similar manner (see additional file 3 or ). The t-test p-value for NHEK 24 hrs is not included, since there were no treatment replicates; an error model was used instead. However, some of the values in the error model do not correspond, in this case, to other t-test p-values and are therefore not included.

**Figure 3:**
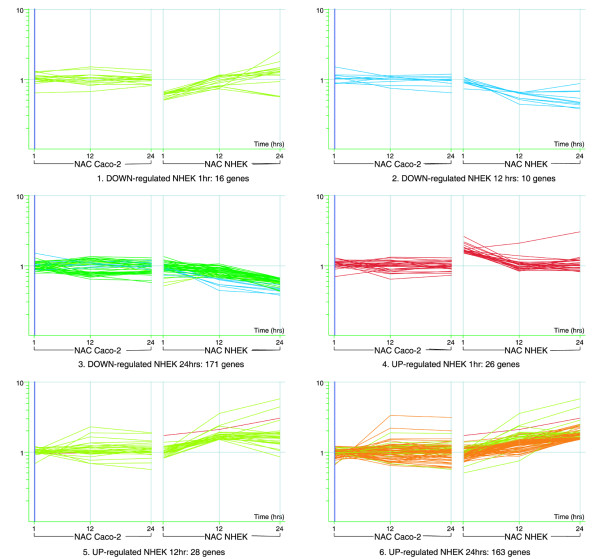
**Matrix view of significantly affected genes. **Genes induced/repressed in NHEK relative to control. The color is specific for genelist of affected genes at each time point (unless these are the same genes as from one of the earlier time points). Also in these cells, quite a limited early response is observed at 1 hr (16 and 26 genes in NHEK). There are almost no genes induced/repressed at 1 hr that continue the trend at later time points -indicating an early transient response. This is markedly different for 12 hrs and 24 hrs treatments that show many common genes. It is also clear that many genes affected by NAC treatment in one cell line don't show the same response in the other cell line.

A similar trend to that described for Caco-2 takes place in these cells, with early transient response at 1 hr, followed by a more extensive response at later time points (12, 24 hrs) as demonstrated in Figure 3. Again, the genes active at 1 hr do not appear to be active at later time points (except for a few genes in each case). On the other hand, genes active at 12 hrs also appear active in the same direction at 24 hrs. Table 1 includes the exact numbers of induced/repressed genes.

### NHEK vs. Caco-2 analysis

Comparisons of NAC treated Caco-2 and NHEK show that the responses are very different. Only a few genes were regulated similarly in both cells (Table 2).

**Table 2:** Genes common in both Caco-2 and NHEK at the different time points.

Time point	Regulation	Probe ID	Gene
1 hr	repressed	36618_g_at	Inhibitor of DNA binding 1, dominant negative helix-loop-helix protein
12 hrs	repressed		none
24 hrs	repressed	33720_at 1069_at	Putatative 28 kDa protein, Human cyclooxygenase-2 (hCox-2) gene, complete cds
1 hr	induced		none
12 hrs	induced	39809_at 36980_at 1585_at	HMG-box transcription factor 1 proline-rich nuclear receptor coactivator 1 v-erb-b2 erythroblastic leukemia viral oncogene homolog 3 (avian)
24hrs	induced	39248_at	Aquaporin 3

**Figure 4:**
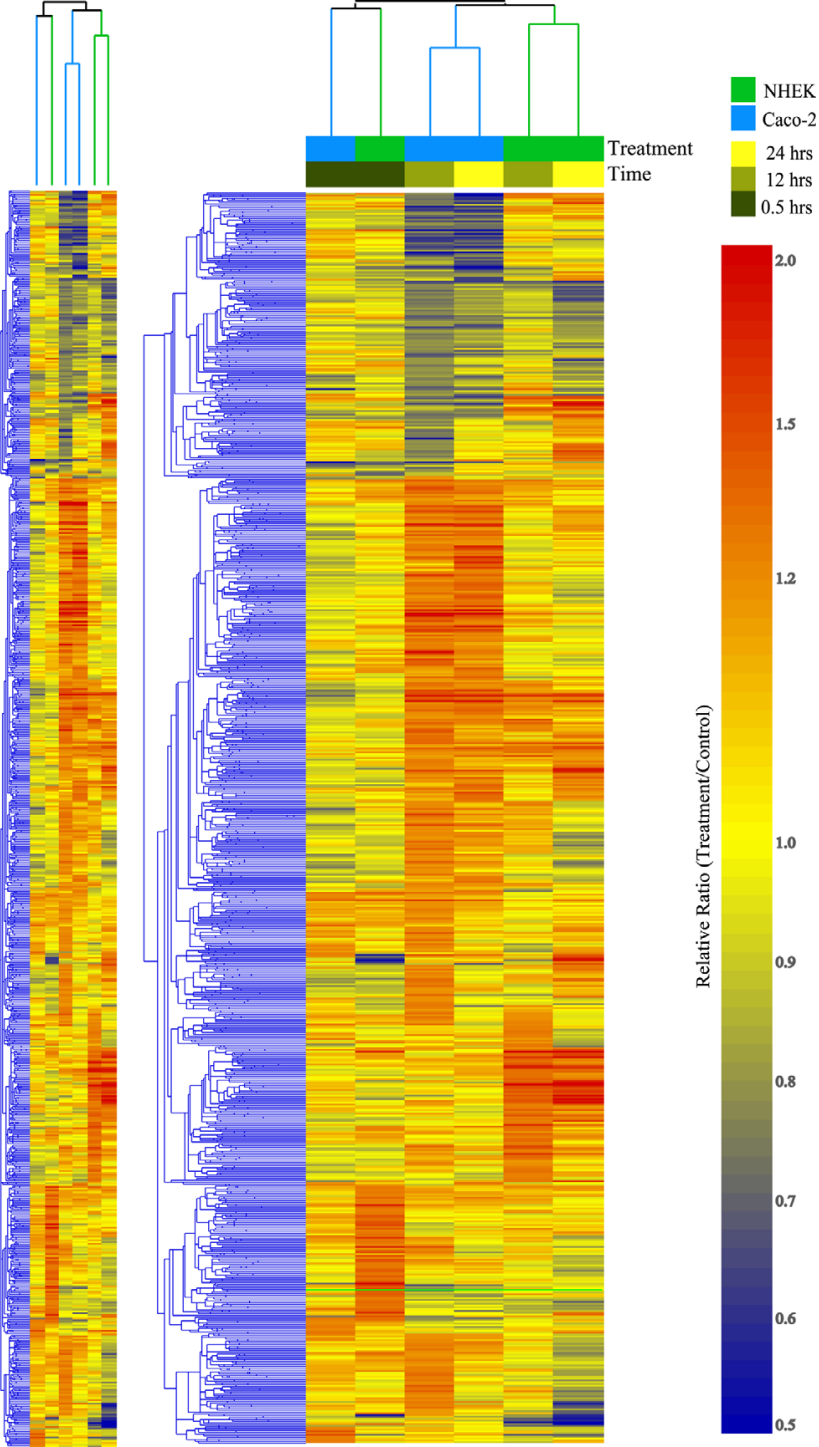
**Hierarchical clustering diagram at 1, 12 and 24 hrs. **A set of 2054 genes which show significant changes in expression in at least one of the time points in either of the two cell line clustered using the standard correlation. Cluster of the corresponding time points for each cell line indicates very close similarity of NHEK and Caco-2 at time point 1 hr, at which the NAC effect is not yet pronounced (both cell lines form a tight separate cluster at 1 hr). However, during the later time points the changes are much more significant and the differences between Caco-2 and NHEK become very pronounced as well (each cell line forms a separate cluster that includes both 12 and 24 hrs time points).

Even if we consider that statistical considerations (filtering etc.) inaccurately prevented a number of genes from overlapping in analysis of two cells, the clustering in Figure 4 show that the responses are indeed very different. The hierarchical clustering is based on filtered genes (significant change in at least one time point) for all time points and each cell type. At the time point 1 h after treatment, there is no cell-specific clustering. Actually, both cell types are in the same cluster for 1 hr. It is in the later time points, with much stronger and distinct response, that the cell-specific clusters form (blue = NHEK, green = Caco-2).

A set of genes indicated to be differentially expressed were chosen to validate the analysis (Table 3). Additional time points were also used in this analysis. The genes represented both markers of proliferation and motility as well as new candidates in these and other related processes and in many of these cases the differential gene expression could be confirmed.

### Gene ontology analysis of Caco-2 and NHEK

In order to perform Gene Ontology analysis of significantly regulated genes in Caco-2 and NHEK we created combined lists of genes up- and down-regulated at any of the three time points. In order to statistically assess the overrepresentation of each category we used the publicly available tool EASE. For each cell line, the down-regulated genes were assessed for gene ontology enrichment relative to the up-regulated genes in the same cell line, within the universe of all genes being the Affymetrix U95aVer2 probe set. The final table of gene ontology groups was selected based on the number of overlapping genes, EASE score, illustrating common biological mechanisms or pathways (e.g. motility, cell division).

The results of analysis in both cell lines indicate a strong tendency towards differentiation by halting cell proliferation and related processes, Table 4 (NHEK) and Table 5 (Caco-2). However, the mechanisms inhibiting cell proliferation in NHEK and Caco-2 cell lines appear to differ significantly. As seen from the two tables, genes responsible for proliferation are shut down in both cell lines, with NHEK showing a stronger direct proliferation effect (more genes are shut down). This effect, however, can be explained by a considerably higher number of significantly regulated genes identified in NHEK vs. Caco-2. Furthermore, the mechanism of inhibiting proliferation in NHEK appears to be directly related to the shut down of processes such as nuclear division, mitosis, cytokinesis etc. – all required for cells to divide and proliferate. In Caco-2, on the other hand, the mechanism appears to involve cell structure inhibition, through the shut down of genes involved in adhesion and cytoskeleton changes. Finally, the motility processes critical to Caco-2 inhibition of proliferation are affected in NHEK, while cell division seems to be affected to a lesser degree in Caco-2.

**Table 4:** Gene ontology for differentially expressed genes found in NHEK cells

**Gene Category**	**Down (genes)**	**Up (genes)**	**Combined list Affy probes**	**EASE score**
**mitotic cell cycle**	41	4	49	1.96E-14
**cell cycle**	52	12	76	2.03E-12
**M phase**	22	1	24	7.73E-09
**DNA replication and chromosome cycle**	22	1	24	7.73E-09
**cell proliferation**	56	22	100	0.000000024
**nuclear division**	20	1	22	7.23E-08
**M phase of mitotic cell cycle**	19	1	21	0.000000217
**DNA replication**	19	1	21	0.000000217
**S phase of mitotic cell cycle**	19	1	21	0.000000217
**mitosis**	19	1	21	0.000000217
**DNA metabolism**	29	6	41	0.000000403
**DNA dependent DNA replication**	13	0	13	0.00000641
**cytokinesis**	12	0	12	0.0000199
metabolism	100	79	256	0.000126
regulation of cell cycle	26	11	48	0.00137
cell growth and/or maintenance	79	62	202	0.00241
nucleobase\, nucleoside\, nucleotide and nucleic acid metabolism	53	36	124	0.00262
biosynthesis	15	5	24	0.00475
protein metabolism	43	29	101	0.00992
physiological process	132	130	389	0.0103
obsolete biological process	17	7	31	0.0125
macromolecule biosynthesis	11	4	18	0.0267
protein modification	22	14	50	0.0639
cytoplasm organization and biogenesis	12	6	23	0.0693
cell organization and biogenesis	17	13	42	0.211
phosphorus metabolism	16	12	40	0.244
phosphate metabolism	16	12	40	0.244
protein amino acid phosphorylation	12	9	30	0.326
phosphorylation	12	10	32	0.424
macromolecule catabolism	10	8	26	0.435
protein catabolism	10	8	26	0.435
proteolysis and peptidolysis	10	8	26	0.435
*cell adhesion*	4	16	36	1
*cell motility*	2	6	14	0.995

**Bold: **processes significantly affected in Caco-2 pointing to differentiation

*Italicized: *processes significantly downregulated in NHEK, pointing to differentiation

Cutoff (EASE score < 5.00E-01)

Cutoff (#overlapping genes > 9, larger than

in Caco-2 due to a large initial list size).

**Table 5:** Gene ontology for differentially expressed genes found in Caco-2 cells

**Gene Category**	**Down (genes)**	**Up (genes)**	**Combined list Affy probes**	**EASE score**
**cell motility**	8	1	10	0.0459
**cytoplasm organization and biogenesis**	8	1	10	0.0459
**muscle development**	7	1	9	0.0872
**morphogenesis**	18	7	33	0.0879
**development**	22	10	43	0.107
**cell adhesion**	11	3	18	0.112
**cell surface receptor linked signal transduction**	11	3	18	0.112
**organelle organization and biogenesis**	6	1	8	0.158
**cytoskeleton organization and biogenesis**	6	1	8	0.158
**cell organization and biogenesis**	11	4	19	0.159
**organogenesis**	16	7	31	0.179
**response to stress**	8	3	14	0.282
**macromolecule biosynthesis**	9	4	17	0.331
**cell-cell signaling**	6	2	10	0.346
signal transduction	19	5	30	0.0118
immune response	9	1	11	0.0233
protein metabolism	15	4	23	0.0235
cellular process	58	34	126	0.0316
defense response	10	2	14	0.0467
transcription\, DNA-dependent	17	10	37	0.358
transcription	17	10	37	0.358
response to biotic stimulus	10	5	20	0.374
nucleobase\, nucleoside\, nucleotide and nucleic acid metabolism	24	16	56	0.434
regulation of transcription\, DNA-dependent	16	10	36	0.442
regulation of transcription	16	10	36	0.442
cell cycle	9	5	19	0.486
*nucleotide metabolism*	3	2	7	0.835
*nuclear division*	3	3	9	0.927
*mitosis*	3	3	9	0.927
*mitotic cell cycle*	5	3	11	0.691
*cytokinesis*	2	2	6	0.952

**Bold: **processes significantly downregulated in NHEK pointing to differentiation

*Italicized: *specific processes significantly downregulated in Caco-2, pointing to differentiation

Cutoff (EASE score) < 5.00E-01

Cutoff (Number of overlapping genes > 5

## Discussion

Deregulation of proliferation is a characteristic of tumorigenesis and therapeutic approaches for cancer treatment targets apoptosis, cell cycle arrest and differentiation. NAC has been shown to induce a multitude of molecular changes related to tumorigenesis [4]. Recently, NAC has been demonstrated to inhibit apoptosis [5,6], possess anti-inflammatory activities [7] and inhibit proliferation [8].

Here, we have monitored the reflection in global gene expression profiles of the transition from proliferation to a differentiated state in normal and cancer cells *in vitro*, as induced by NAC. Two out of three previous studies of the global gene expression that accompanies the spontaneous differentiation of Caco-2 report a general down-regulation of gene expression in differentiated cells as compared to the proliferating counterpart [9-11]. A similar, but not as pronounced, trend is reflected in the number of genes differentially expressed following NAC induced differentiation in Caco-2.

The expression level of 253 targets in Caco-2 and 414 in NHEK were statistically differentially expressed at different time points. Multiple appearances of differentially regulated transcripts were common, resulting in detection of totally less than 200 unique genes, respectively. This is fewer than expected in comparison to previous reports on differential regulation during spontaneous Caco-2 differentiation and probably due to the difference in stringency of the algorithms used for data analyses (MAS 4.0 vs RMA), rather than related to functional biological discrepancies.

### Initial response

In both data sets, the early responses were relatively limited and appeared to be transient, indicating that a large part of initial immediate early events occur at the level of translation and post-translational modifications. The nature of initial regulatory events is also expected to be transient due to feedback inhibition as well as to a restricted number of NAC induced mechanisms.

Interestingly, significant transcriptional down-regulation of the *inhibitor of differentiation 1 (ID-1) *was found at 1 hr in both Caco-2 and NHEK, suggesting a common mechanism of NAC induced differentiation and inhibition of proliferation in NHEK and Caco-2 epithelial cells. Analysis of ID-1 expression levels by real-time quantitative PCR confirmed the suppression of the transcript in both cell types (Table 3). ID-1 has been demonstrated to bind helix-loop helix transcription factors, preventing them from binding DNA [12]. In particular, ID-1 has been shown to be required for G1 progression, and its constitutive expression inhibited the lineage commitment and differentiation in B-cells [13,14]. Previous reports have also shown that ID-1 is a negative transcriptional regulator of *CDKN2A *(*p16/p14/p19*), which induces G1 arrest through the inhibition of Rb phosphorylation by cdk -4 and -6 [15]. Overexpression of *ID-1 *was also reported in psoriatic involved skin [16]. Inhibitors of histone deacetylase activity are emerging as a potentially important new class of anticancer agents. The cell cycle blockade and differentiation caused by such a drug, trichostatin A, caused decreased levels of ID-1 consistent with cell cycle senescence and differentiation of A2780 ovarian cancer cells [17]. Vitamin D is also known to promote differentiation and was shown by others to down-regulate *ID-1 *through a suppressive vitamin D response sequence in the 5'of the gene [18]. The *ID-1 *expression is regulated by a protein complex containing the immediate-early response gene *EGR1 *[19].

The growth regulatory properties of EGR1 have been found to involve coordinated regulation of TGF-β_1 _and fibronectin (FN1). The resulting proteins are secreted and lead to increased expression of plasminogen activator inhibitor-1 (PAI1). Both the secreted FN1 and PAI1 functions to enhance cell attachment and normal cell growth [20]. We detect the induction of both fibronectin and PAI1 in NHEK cells at both 12 and 24 hrs, suggesting a role of EGR1 pathways in the NAC mediated mechanism at least in this cell type.

Other interesting down-regulated genes at 1 h after NAC treatment in NHEK included for example *regulated in development and DNA damage response 1 *(*REDD1*), *squamous cell carcinoma antigen 1 *(*SCCA1*), *highly expressed in cancer *(*HEC*), *s100a9 *and *kallikrein 7 *(also termed *stratum corneum chymotryptic enzyme *– *SCCE*). *REDD1 *has previously been shown to be down-regulated in differentiating primary human keratinocytes and ectopic expression inhibits in vitro differentiation [21]. The suppression of *SCCA1 *has been demonstrated to inhibit tumour growth [22], *HEC *expression is increased in tumours [23] and *S100A9 *has been found to be up-regulated in psoriasis patients displaying keratinocyte hyperproliferation and altered differentiation [24]. *SCCE *has been suggested to play a role in desquamation and its up-regulation is associated with poor prognosis of ovarian and breast cancer [25,26]. A number of interesting up-regulated genes, such as *activin A *and *WEE1*, were also identified in NHEK 1 hr after NAC treatment. Over expression of WEE1 inhibits cell cycle progression by inactivation of the CDC2/cylin B complex [27], while a*ctivin A *is a member of the TGF-β family of cytokines which is known to promote growth arrest and differentiation in several tissues including intestinal epithelia [28,29]. In fact it was first identified as a protein that exhibits a potent differentiation-inducing activity [30]. In Caco-2, the proto-oncogene *fos *and the transcription factor *HNF3A*, which have been observed to be amplified in human malignancies [31], was two of the identified genes being repressed at 1 hr after NAC treatment. A single gene, *integrin alpha 2*, was found up-regulated at this time-point.

It is clear that these data collectively describe molecular changes associated with the mediation of a differentiated epithelial phenotype. A large part of the differentially expressed genes have clear implications in withdrawal of mitogenic signals and in promotion of growth arrest. Multiple signalling pathways are suggested to be involved.

### Late response (multiple occurrences)

Progressively more genes were affected in both cell types, and many showed similar trends in direction, over later time points. Cluster analysis revealed tightly linked genes between the 12 and 24 hrs time points in the same cell type and genes with such multiple appearances are potentially more strongly implicated in the differentiation process.

In NHEK cells, for example the down-regulated mitogens *neuregulin 1 *(*heregulin*) and *melanoma growth stimulatory activity *(*MGSA*) [32], belong to this group. *MGSA *belongs to a super family of chemochines, including *IL-8*, which is involved in inflammatory processes. Heregulin is known to activate the oncogenic ERBB2 receptor [33]. Cdc-6, which are regulated in response to mitogenic signals, binds PCNA and is required for initiation of DNA replication [34], was also repressed at both 12 and 24 hrs after NAC treatment, implying programs involving withdrawal of mitogenic factors as important mechanisms for NAC mediated inhibition of proliferation and increased differentiation in NHEK cells. The expression of *Topoisomerase II *(*TOP2*) was also repressed, confirming results obtained in NAC treated CHO cells [35]. Topoisomerases control and alter the topologic states of DNA, and the relaxation activity of TOP2 is essential for productive RNA synthesis on nucleosomal DNA [36].

The list of correspondingly important up-regulated genes in NHEK was extensive and included *activin A*, *transglutaminase 2 *(*TGM2*), *ErbB3 *(*HER3*), *matrix metalloproteinase 9 *(*MMP-9*), *fibronectin (FN!)*, *PAI1 *and *TGF*β among others. Notably, *activin A *was the only gene found to be up-regulated at all investigated time points, demonstrating a sustained growth inhibitory and differentiation promoting signal. TGM2 catalyses cross-linking of proteins, demonstrates G-protein function in receptor signalling [37] and was recently reported to phosphorylate IGFB3 [38]. IGFB3 in turn has a major role in regulation of proliferation as a growth inhibitor through IGF2 binding and alternative IGF2 independent pathways [39]. Thus implicating potential regulatory functions of *TGM2 *in proliferation and differentiation. Surprisingly, *ErbB3*, which promotes proliferation through the Wnt signalling pathway, was also up-regulated. RT-PCR could confirm the induction (Caco-2, 12 hrs) and a study investigating spontaneous Caco-2 differentiation is also in agreement with our data on up-regulation [11]. In contrast, a recent publication reported its up-regulation in breast cancer [40], suggesting a dual role of ERBB3 in cell cycle regulation. The induction of *MMP9*, confirmed by RT-PCR (48 h NHEK) is in additional contrast to our observations of NAC-induced cell differentiation and proliferation. MMP9 has been associated with angiogenesis, tumour progression and metastasis as mediated through degradation of the extracellular matrix (ECM) [41] and stimulation of hyperproliferation [42]. However, tumours with low levels of MMP9 were found to be less differentiated. Thus, although MMP9 stimulates proliferation, it is also implied in positive regulation of differentiation [42]. NAC has been proposed to inhibit activation of latent MMP9 protein in ECM reservoirs by removal of its propeptide and by competing for the zink ion which is necessary for enzymatic function [43]. Hence, our data may imply that post-transcriptional regulation of MMP9 prevails over the transcriptional changes as the major control mechanism.

A large number of multiple occurring differentially expressed transcripts were demonstrated in Caco-2, including *intestinal trefoilfactor 3 *(*TFF3*) and *Aquaporin 3 *(*AQP3*) among others. TFF3 has been shown to have a central role in the maintenance and repair of intestinal mucosa [44] and upregulation is expected during differentiation. Aquaporins (AQPs) are water channel proteins, important for the transport of water and other small proteins across the cell membrane [45]. *AQP1 *has previously been shown to be involved in cell cycle control [46], suggesting that *AQP3 *may also have a role in the progression of cancer. *AQP3 *has been reported to be highly expressed in several types of stratified epithelial cells in rat, including the epidermis [47] and the differentiated cells of the gastrointestinal tract [48]. The expression of *AQP3 *was reported to be up-regulated in differentiating Caco-2 cells [11], while expression was shown to be down-regulated in differentiating primary keratinocytes [49]. In this study AQP appears to have a transient behaviour in Caco-2 cells with a repressed behaviour at initial time point and induced pattern at later time points (confirmed by RT-PCR), indicating a remodelling of cell membrane constituents.

The genes repressed in Caco-2 included for example *Cyclin D1*, *Inhibin beta B*, *BMP-2 *and *FHL-2*. The D1 cyclin is involved in β-catenin-TCF signalling and its down-regulation induce G1 arrest [50]. FHL-2 has been demonstrated to be a coactivator of β-catenin from cyclin D and IL-8 promoters in a colon cell line [51], suggesting that repression of FHL-2 may also repress growth. *Inhibin beta *and *BMP-2 *are members of the *TGF *family of genes. Inhibin beta is an antagonist of activin A activity and consequently represses differentiation and promotes growth [52]. *BMP-2 *on the other hand, has been demonstrated both to induce apoptosis [53] and growth inhibition/differentiation [54]. In contrast, another recent study demonstrated the ability of *BMP-2 *to enhance the growth of tumours [55].

#### Late response (single occurrence)

A vast number of additional interesting genes with potentially important roles in mediation and manifestation of the differentiated epithelial phenotype was identified as significantly induced or repressed in a single time point.

As an example, up-regulation in NHEK after 24 hrs was seen for transcripts encoding *CDKN2B *(*p15*), which is believed to be an effector of TGF-β induced G1 arrest and inhibition of proliferation [56], and for *CDKN1C*, which is a *p21 *homologue and negative regulator of cell proliferation [57]. Other up-regulated genes at this time point were the transcription factor *Jun *and *BTG1*. *Jun *was recently demonstrated to be a regulator of erythroid differentiation [58] and *Jun B *knock out mice have been shown to develop a proliferative disease resembeling human chronic myeloid leukemia [59]. *BTG1 *has been proposed to belong to a family of antiproliferative genes [60]. Up-regulation was also found for *cadherin 13*, a gene with growth inhibitory functions that is expressed in normal cells but not in the majority of human tumour cells of epithelial origin [61]. While not identified by global transcript analysis, RT-PCR investigation revealed increased levels of *E-cadherin *in both Caco-2 (24 hrs) and NHEK (24 and 48 hrs), this in agreement with the previously reported immunohistochemical data [1] that showed increased staining of E-cadherin in NAC treated cells. In Caco-2 cells, the differentiation-related gene *NDRG1*, which is expressed during differentiation and down-regulated in colorectal neoplasms [62], was up-regulated at 12 hrs. This increase was also demonstrated by RT-PCR at 12, 24 and 48 hrs after NAC treatment. The induction of *Cdx2 *may also be a functional change, since reduced expression of *Cdx2 *has been shown to be important in colon tumorigenesis [63]. Interestingly, in correlation with the controversial results from NHEK, an up-regulation of *ErbB3 *was identified in Caco-2 at 12 hrs. In addition, the oncogene *myc *was also up-regulated in contrast to the expected decrease. The down-regulation of C*ox-2 *and *BMP-2 *was also in concordance with NHEK data. In addition, the TGF-β family member *BMP-4 *was repressed. This correlates well with our results on repressed *ID-1 *expression, since both BMP-2 and -4 up-regulate *ID-1 *[64,65].

Hence, although induced by the same mechanism (NAC) and yielding the same end-stage of growth inhibition and differentiation, the processes in NHEK and Caco-2 are on the whole quite different. This is demonstrated by gene specific differences that result in lack of correlation between cell types at the same time point after treatment as identified by cluster analysis. This is furthermore supported by the Gene Ontology analysis, tables 4 and 5, indicating that the two cell lines achieve their differentiated states using two distinct mechanisms, this in concordance with previously observed effects of NAC treatment on cell morphology and growth arrest [1]. It should be noted that apoptosis does not appear to act as a regulating mechanism, since only a very small proportion of apoptotic genes are affected in either direction (2 to 12 genes out of 317, in either direction). This is also supported by the previous study [1], which analysed apoptosis by propidium iodide labelling and flow cytometry.

As additional testimony to the lineage specific differentiation programs, only very few genes were identified as being similarly regulated in both cell-types. These included *ID-1*, *AQP3 *and *ErbB3 *(as described above). *Cox-2 *was also similarly down-regulated in both cell types after NAC treatment. When investigating *Cox-2 *expression by RT-PCR we could confirm the down-regulation at 24 hrs in Caco-2 and identify an additional repression in NHEK at 1, 3 and 48 hrs after NAC treatment. Overexpression of *Cox-2 *has been shown to promote cell migration and invasion in Caco-2 cells [66] and to regulate colon carcinoma induced angiogenesis by production of angiogenic factors [67]. In addition, epidermal differentiation is also affected by *Cox-2 *over expression. *Cox-2 *seems to prevent entrance into the postmitotic state, which is coupled to the switching on expression of differentiation-associated proteins, allowing keratinocytes to proliferate [68]. In correlation with our data, a NAC mediated inhibition of *Cox-2 *expression have previously been demonstrated in colorectal cancer [69]. Hence, it is likely that *Cox-2 *repression is a NAC specific event endorsing differentiation/growth arrest in both NHEK and Caco-2. *HBP1 *was also up-regulated in both Caco-2 and NHEK after 12 h of NAC treatment and RT-PCR could confirm an increase in Caco-2 at 12 and 24 hrs. This corresponds to previous findings, where *HBP1 *has been seen to have a negative effect on tumours. It has previously been established that *HBP1 *is a target of the retinoblastoma pathways [70,71] and that *HBP1 *negatively regulates Wnt/β-catenin, thus inhibiting proliferation and suggesting that *HBP1 *may have a tumour suppressor function [72]. Two additional proteins, *putative 28 kDa protein *and *proline rich nuclear receptor coactivator-1 *(*PNRC1*), were also identified as differentially expressed in both cell types. RT-PCR analysis was able to confirm an up-regulation of *PNRC1 *in Caco-2 24 hrs after treatment. However, these genes are not previously described and potential functions remain unresolved.

A proportion of the differentially expressed transcripts were not possible to predict as being part of the differentiation context. For example, *ErbB3*, *fos*, *TGFβI *and *myc *were found to be expressed at higher levels in differentiated cells, in contrast to their roles in promotion of proliferation. Interestingly, a similar contrasting increase in *fos *and *ErbB3 *levels was found in normal colonic cells as compared to colorectal cancers in a SAGE study [73]. It is important to note that spontaneous morphological and functional differentiation in Caco-2 have been demonstrated not to be coupled, with independent mosaic patterns of proliferating and differentiated cells present adjacent in the cell culture [74], which may in part explain some of the contrasting results. However, many of the detected differentially expressed genes in this study have previously been described as altered in differentiated epithelial cells. In NHEK cells we could for example confirm the expected increased expression of *SPRR1B *and *SPRR2A*. In Caco-2 the up-regulation of *AQP3*, *NDRG1 *and *TFF3 *were among the genes that validated the results from the global transcription profile analysis.

Interestingly, several genes with major relevance in psoriasis have been found differentially expressed as a consequence of NAC treatment in the particular epithelial cells included in this study, for example *S100A9*, *ID-1 and Cox-2*. These findings could give a mechanistic background to the ongoing clinical studies being carried out based on empirical NAC treatment of patients having psoriasis.

Alternative NAC signalling mechanisms at the level of proteins and metabolites may also be important. For instance the phospholipid modulator, *platelet activating factor*, has been demonstrated to induce differentiation and inhibit proliferation in colon cells [75], and inhibit proliferation in cultured human keratinocytes [76]. Accordingly, we are performing supplementary proteome and metabolome studies. In addition, analyses of additional cell lines for finding a common pathway of molecular changes that result from NAC induced differentiation are being considered.

## Conclusion

Our data demonstrate that NAC stimulated differentiation induces a limited and transient early transcriptional change, followed by a more constitutive and extensively different expression at later time points in both NHEK and Caco-2 cells. The genes affected are to a large extent related to inhibition of proliferation and stimulation of differentiation, but the responses are almost completely lineage specific. This and further analysis of NAC mediated expression changes provide a description of the complex molecular mechanisms of sulphydryl reductant treatment and potential targets for the development of new drugs for treatment of proliferation related epithelial disorders.

## List of abbreviations

Inhibitor of differentiation 1 (ID-1)

N-acetyl-L-cysteine (NAC)

Normal human epidermal keratinocytes (NHEK)

Glyceraldehyde-3-phosphate dehydrogenase (GAPDH)

Transferrin receptor (TFR)

Fibronectin (FN1)

Plasminogen activator inhibitor-1 (PAI1)

Regulated in development and DNA damage response 1 (REDD1),

Squamous cell carcinoma antigen 1 (SCCA1)

Highly expressed in cancer (HEC)

Stratum corneum chymotryptic enzyme (SCCE)

Melanoma growth stimulatory activity (MGSA)

Topoisomerase II (TOP2)

Transglutaminase 2 (TGM2)

Matrix metalloproteinase 9 (MMP-9)

Intestinal trefoilfactor 3 (TFF3)

Aquaporin 3 (AQP3)

Proline rich nuclear receptor coactivator-1 (PNRC1)

## Competing interests

The author(s) declare that they have no competing interests.

## Authors' contributions

ACG participated in the design of the study, drafted the manuscript, coordinated and carried out real-time kinetic PCR and Affymetrix experiments as well as performed initial data processing and data analysis. IK performed data processing, statistical analysis and data analysis as well as assisted with the manuscript. EER performed real-time kinetic PCRs and assisted in drafting the manuscript. LBL, GG and EKK cultured cells and isolated totRNA. JL, TP, TL and MCR directed the teams that carried out this study.

## Pre-publication history

The pre-publication history for this paper can be accessed here:



## Supplementary Material

Additional File 1 Summary of the top 10 differentially expressed genes in Caco-2, at all time points studied. Click here for file

Additional File 2 Summary of the top 10 differentially expressed genes in NHEK, at all time points studied. Click here for file

Additional File 3 All differentially expressed transcripts in NHEK at all time points. Click here for file

Additional File 4 All differentially expressed transcripts in Caco-2 at all time points. Click here for file

## References

[B1] Parasassi T, Brunelli R, Bracci-Laudiero L, Greco G, Gustafsson AC, Krasnowska EK, Lundeberg J, Lundeberg T, Pittaluga E, Romano MC, Serafino A (2005). Differentiation of normal and cancer cells induced by sulfhydryl reduction: biochemical and molecular mechanisms. Cell Death Differ.

[B2] Delie F, Rubas W (1997). A human colonic cell line sharing similarities with enterocytes as a model to examine oral absorption: advantages and limitations of the Caco-2 model. Crit Rev Ther Drug Carrier Syst.

[B3] Hosack DA, Dennis G, Sherman BT, Lane HC, Lempicki RA (2003). Identifying biological themes within lists of genes with EASE. Genome Biol.

[B4] De Flora S, Izzotti A, D'Agostini F, Balansky RM (2001). Mechanisms of N-acetylcysteine in the prevention of DNA damage and cancer, with special reference to smoking-related end-points. Carcinogenesis.

[B5] Rosati E, Sabatini R, Ayroldi E, Tabilio A, Bartoli A, Bruscoli S, Simoncelli C, Rossi R, Marconi P (2004). Apoptosis of human primary B lymphocytes is inhibited by N-acetyl-L-cysteine. J Leukoc Biol.

[B6] Hart AM, Terenghi G, Kellerth JO, Wiberg M (2004). Sensory neuroprotection, mitochondrial preservation, and therapeutic potential of n-acetyl-cysteine after nerve injury. Neuroscience.

[B7] Rhoden CR, Lawrence J, Godleski JJ, Gonzalez-Flecha B (2004). N-acetylcysteine prevents lung inflammation after short-term inhalation exposure to concentrated ambient particles. Toxicol Sci.

[B8] Estensen RD, Levy M, Klopp SJ, Galbraith AR, Mandel JS, Blomquist JA, Wattenberg LW (1999). N-acetylcysteine suppression of the proliferative index in the colon of patients with previous adenomatous colonic polyps. Cancer Lett.

[B9] Tadjali M, Seidelin JB, Olsen J, Troelsen JT (2002). Transcriptome changes during intestinal cell differentiation. Biochim Biophys Acta.

[B10] Mariadason JM, Arango D, Corner GA, Aranes MJ, Hotchkiss KA, Yang W, Augenlicht LH (2002). A gene expression profile that defines colon cell maturation in vitro. Cancer Res.

[B11] Fleet JC, Wang L, Vitek O, Craig BA, Edenberg HJ (2003). Gene expression profiling of Caco-2 BBe cells suggests a role for specific signaling pathways during intestinal differentiation. Physiol Genomics.

[B12] Sun XH, Copeland NG, Jenkins NA, Baltimore D (1991). Id proteins Id1 and Id2 selectively inhibit DNA binding by one class of helix-loop-helix proteins. Mol Cell Biol.

[B13] Hara E, Yamaguchi T, Nojima H, Ide T, Campisi J, Okayama H, Oda K (1994). Id-related genes encoding helix-loop-helix proteins are required for G1 progression and are repressed in senescent human fibroblasts. J Biol Chem.

[B14] Sun XH (1994). Constitutive expression of the Id1 gene impairs mouse B cell development. Cell.

[B15] Ohtani N, Zebedee Z, Huot TJ, Stinson JA, Sugimoto M, Ohashi Y, Sharrocks AD, Peters G, Hara E (2001). Opposing effects of Ets and Id proteins on p16INK4a expression during cellular senescence. Nature.

[B16] Bjorntorp E, Parsa R, Thornemo M, Wennberg AM, Lindahl A (2003). The helix-loop-helix transcription factor Id1 is highly expressed in psoriatic involved skin. Acta Derm Venereol.

[B17] Strait KA, Dabbas B, Hammond EH, Warnick CT, Iistrup SJ, Ford CD (2002). Cell cycle blockade and differentiation of ovarian cancer cells by the histone deacetylase inhibitor trichostatin A are associated with changes in p21, Rb, and Id proteins. Mol Cancer Ther.

[B18] Ezura Y, Tournay O, Nifuji A, Noda M (1997). Identification of a novel suppressive vitamin D response sequence in the 5'-flanking region of the murine Id1 gene. J Biol Chem.

[B19] Tournay O, Benezra R (1996). Transcription of the dominant-negative helix-loop-helix protein Id1 is regulated by a protein complex containing the immediate-early response gene Egr-1. Mol Cell Biol.

[B20] Liu C, Yao J, de Belle I, Huang RP, Adamson E, Mercola D (1999). The transcription factor EGR-1 suppresses transformation of human fibrosarcoma HT1080 cells by coordinated induction of transforming growth factor-beta1, fibronectin, and plasminogen activator inhibitor-1. J Biol Chem.

[B21] Ellisen LW, Ramsayer KD, Johannessen CM, Yang A, Beppu H, Minda K, Oliner JD, McKeon F, Haber DA (2002). REDD1, a developmentally regulated transcriptional target of p63 and p53, links p63 to regulation of reactive oxygen species. Mol Cell.

[B22] Suminami Y, Nagashima S, Murakami A, Nawata S, Gondo T, Hirakawa H, Numa F, Silverman GA, Kato H (2001). Suppression of a squamous cell carcinoma (SCC)-related serpin, SCC antigen, inhibits tumor growth with increased intratumor infiltration of natural killer cells. Cancer Res.

[B23] Chen Y, Riley DJ, Chen PL, Lee WH (1997). HEC, a novel nuclear protein rich in leucine heptad repeats specifically involved in mitosis. Mol Cell Biol.

[B24] Semprini S, Capon F, Tacconelli A, Giardina E, Orecchia A, Mingarelli R, Gobello T, Zambruno G, Botta A, Fabrizi G, Novelli G (2002). Evidence for differential S100 gene over-expression in psoriatic patients from genetically heterogeneous pedigrees. Hum Genet.

[B25] Yousef GM, Diamandis EP (2003). Tissue kallikreins: new players in normal and abnormal cell growth?. Thromb Haemost.

[B26] Talieri M, Diamandis EP, Gourgiotis D, Mathioudaki K, Scorilas A (2004). Expression analysis of the human kallikrein 7 (KLK7) in breast tumors: a new potential biomarker for prognosis of breast carcinoma. Thromb Haemost.

[B27] McGowan CH, Russell P (1993). Human Wee1 kinase inhibits cell division by phosphorylating p34cdc2 exclusively on Tyr15. Embo J.

[B28] Sonoyama K, Rutatip S, Kasai T (2000). Gene expression of activin, activin receptors, and follistatin in intestinal epithelial cells. Am J Physiol Gastrointest Liver Physiol.

[B29] Chen YG, Lui HM, Lin SL, Lee JM, Ying SY (2002). Regulation of cell proliferation, apoptosis, and carcinogenesis by activin. Exp Biol Med (Maywood).

[B30] Murata M, Eto Y, Shibai H, Sakai M, Muramatsu M (1988). Erythroid differentiation factor is encoded by the same mRNA as that of the inhibin beta A chain. Proc Natl Acad Sci U S A.

[B31] Lin L, Miller CT, Contreras JI, Prescott MS, Dagenais SL, Wu R, Yee J, Orringer MB, Misek DE, Hanash SM (2002). The hepatocyte nuclear factor 3 alpha gene, HNF3alpha (FOXA1), on chromosome band 14q13 is amplified and overexpressed in esophageal and lung adenocarcinomas. Cancer Res.

[B32] Horuk R, Yansura DG, Reilly D, Spencer S, Bourell J, Henzel W, Rice G, Unemori E (1993). Purification, receptor binding analysis, and biological characterization of human melanoma growth stimulating activity (MGSA). Evidence for a novel MGSA receptor. J Biol Chem.

[B33] Holmes WE, Sliwkowski MX, Akita RW, Henzel WJ, Lee J, Park JW, Yansura D, Abadi N, Raab H, Lewis GD (1992). Identification of heregulin, a specific activator of p185erbB2. Science.

[B34] Yan Z, DeGregori J, Shohet R, Leone G, Stillman B, Nevins JR, Williams RS (1998). Cdc6 is regulated by E2F and is essential for DNA replication in mammalian cells. Proc Natl Acad Sci U S A.

[B35] Grdina DJ, Murley JS, Roberts JC (1998). Effects of thiols on topoisomerase-II alpha activity and cell cycle progression. Cell Prolif.

[B36] Mondal N, Parvin JD (2001). DNA topoisomerase IIalpha is required for RNA polymerase II transcription on chromatin templates. Nature.

[B37] Fesus L, Piacentini M (2002). Transglutaminase 2: an enigmatic enzyme with diverse functions. Trends Biochem Sci.

[B38] Mishra S, Murphy LJ (2004). Tissue transglutaminase has intrinsic kinase activity: Identification of transglutaminase 2 as an insulin-like growth factor binding protein-3 kinase. J Biol Chem.

[B39] Ricort JM, Binoux M (2002). Insulin-like growth factor-binding protein-3 activates a phosphotyrosine phosphatase. Effects on the insulin-like growth factor signaling pathway. J Biol Chem.

[B40] Perez-Nadales E, Lloyd AC (2004). Essential function for ErbB3 in breast cancer proliferation. Breast Cancer Res.

[B41] Ray JM, Stetler-Stevenson WG (1994). The role of matrix metalloproteases and their inhibitors in tumour invasion, metastasis and angiogenesis. Eur Respir J.

[B42] Coussens LM, Werb Z (1996). Matrix metalloproteinases and the development of cancer. Chem Biol.

[B43] Cai T, Fassina G, Morini M, Aluigi MG, Masiello L, Fontanini G, D'Agostini F, De Flora S, Noonan DM, Albini A (1999). N-acetylcysteine inhibits endothelial cell invasion and angiogenesis. Lab Invest.

[B44] Mashimo H, Wu DC, Podolsky DK, Fishman MC (1996). Impaired defense of intestinal mucosa in mice lacking intestinal trefoil factor. Science.

[B45] Agre P, Brown D, Nielsen S (1995). Aquaporin water channels: unanswered questions and unresolved controversies. Curr Opin Cell Biol.

[B46] Delporte C, Chen ZJ, Baum BJ (1996). Aquaporin 1 expression during the cell cycle in A5 cells. Biochem Biophys Res Commun.

[B47] Matsuzaki T, Suzuki T, Koyama H, Tanaka S, Takata K (1999). Water channel protein AQP3 is present in epithelia exposed to the environment of possible water loss. J Histochem Cytochem.

[B48] Koyama Y, Yamamoto T, Tani T, Nihei K, Kondo D, Funaki H, Yaoita E, Kawasaki K, Sato N, Hatakeyama K, Kihara I (1999). Expression and localization of aquaporins in rat gastrointestinal tract. Am J Physiol.

[B49] Zheng X, Bollinger Bollag W (2003). Aquaporin 3 colocates with phospholipase d2 in caveolin-rich membrane microdomains and is downregulated upon keratinocyte differentiation. J Invest Dermatol.

[B50] Heinen CD, Goss KH, Cornelius JR, Babcock GF, Knudsen ES, Kowalik T, Groden J (2002). The APC tumor suppressor controls entry into S-phase through its ability to regulate the cyclin D/RB pathway. Gastroenterology.

[B51] Wei Y, Renard CA, Labalette C, Wu Y, Levy L, Neuveut C, Prieur X, Flajolet M, Prigent S, Buendia MA (2003). Identification of the LIM protein FHL2 as a coactivator of beta-catenin. J Biol Chem.

[B52] Bernard DJ, Chapman SC, Woodruff TK (2001). Mechanisms of inhibin signal transduction. Recent Prog Horm Res.

[B53] Hallahan AR, Pritchard JI, Chandraratna RA, Ellenbogen RG, Geyer JR, Overland RP, Strand AD, Tapscott SJ, Olson JM (2003). BMP-2 mediates retinoid-induced apoptosis in medulloblastoma cells through a paracrine effect. Nat Med.

[B54] Wen XZ, Miyake S, Akiyama Y, Yuasa Y (2004). BMP-2 modulates the proliferation and differentiation of normal and cancerous gastric cells. Biochem Biophys Res Commun.

[B55] Langenfeld EM, Calvano SE, Abou-Nukta F, Lowry SF, Amenta P, Langenfeld J (2003). The mature bone morphogenetic protein-2 is aberrantly expressed in non-small cell lung carcinomas and stimulates tumor growth of A549 cells. Carcinogenesis.

[B56] Hannon GJ, Beach D (1994). p15INK4B is a potential effector of TGF-beta-induced cell cycle arrest. Nature.

[B57] Lee MH, Reynisdottir I, Massague J (1995). Cloning of p57KIP2, a cyclin-dependent kinase inhibitor with unique domain structure and tissue distribution. Genes Dev.

[B58] Jacobs-Helber SM, Abutin RM, Tian C, Bondurant M, Wickrema A, Sawyer ST (2002). Role of JunB in erythroid differentiation. J Biol Chem.

[B59] Passegue E, Jochum W, Schorpp-Kistner M, Mohle-Steinlein U, Wagner EF (2001). Chronic myeloid leukemia with increased granulocyte progenitors in mice lacking junB expression in the myeloid lineage. Cell.

[B60] Rouault JP, Rimokh R, Tessa C, Paranhos G, Ffrench M, Duret L, Garoccio M, Germain D, Samarut J, Magaud JP (1992). BTG1, a member of a new family of antiproliferative genes. Embo J.

[B61] Lee SW (1996). H-cadherin, a novel cadherin with growth inhibitory functions and diminished expression in human breast cancer. Nat Med.

[B62] van Belzen N, Dinjens WN, Diesveld MP, Groen NA, van der Made AC, Nozawa Y, Vlietstra R, Trapman J, Bosman FT (1997). A novel gene which is up-regulated during colon epithelial cell differentiation and down-regulated in colorectal neoplasms. Lab Invest.

[B63] Aoki K, Tamai Y, Horiike S, Oshima M, Taketo MM (2003). Colonic polyposis caused by mTOR-mediated chromosomal instability in Apc+/Delta716 Cdx2+/- compound mutant mice. Nat Genet.

[B64] Shepherd TG, Nachtigal MW (2003). Identification of a putative autocrine bone morphogenetic protein-signaling pathway in human ovarian surface epithelium and ovarian cancer cells. Endocrinology.

[B65] Langenfeld EM, Langenfeld J (2004). Bone morphogenetic protein-2 stimulates angiogenesis in developing tumors. Mol Cancer Res.

[B66] Tsujii M, Kawano S, DuBois RN (1997). Cyclooxygenase-2 expression in human colon cancer cells increases metastatic potential. Proc Natl Acad Sci U S A.

[B67] Tsujii M, Kawano S, Tsuji S, Sawaoka H, Hori M, DuBois RN (1998). Cyclooxygenase regulates angiogenesis induced by colon cancer cells. Cell.

[B68] Neufang G, Furstenberger G, Heidt M, Marks F, Muller-Decker K (2001). Abnormal differentiation of epidermis in transgenic mice constitutively expressing cyclooxygenase-2 in skin. Proc Natl Acad Sci U S A.

[B69] Chinery R, Beauchamp RD, Shyr Y, Kirkland SC, Coffey RJ, Morrow JD (1998). Antioxidants reduce cyclooxygenase-2 expression, prostaglandin production, and proliferation in colorectal cancer cells. Cancer Res.

[B70] Lavender P, Vandel L, Bannister AJ, Kouzarides T (1997). The HMG-box transcription factor HBP1 is targeted by the pocket proteins and E1A. Oncogene.

[B71] Tevosian SG, Shih HH, Mendelson KG, Sheppard KA, Paulson KE, Yee AS (1997). HBP1: a HMG box transcriptional repressor that is targeted by the retinoblastoma family. Genes Dev.

[B72] Sampson EM, Haque ZK, Ku MC, Tevosian SG, Albanese C, Pestell RG, Paulson KE, Yee AS (2001). Negative regulation of the Wnt-beta-catenin pathway by the transcriptional repressor HBP1. Embo J.

[B73] Zhang L, Zhou W, Velculescu VE, Kern SE, Hruban RH, Hamilton SR, Vogelstein B, Kinzler KW (1997). Gene expression profiles in normal and cancer cells. Science.

[B74] Vachon PH, Beaulieu JF (1992). Transient mosaic patterns of morphological and functional differentiation in the Caco-2 cell line. Gastroenterology.

[B75] Wang H, Chakrabarty S (2003). Platelet-activating factor activates mitogen-activated protein kinases, inhibits proliferation, induces differentiation and suppresses the malignant phenotype of human colon carcinoma cells. Oncogene.

[B76] Shimada A, Ota Y, Sugiyama Y, Sato S, Kume K, Shimizu T, Inoue S (1998). In situ expression of platelet-activating factor (PAF)-receptor gene in rat skin and effects of PAF on proliferation and differentiation of cultured human keratinocytes. J Invest Dermatol.

