# Supplementary material for: Global gene expression analysis in time series following N-acetyl L-cysteine induced epithelial differentiation of human normal and cancer cells in vitro
Source: BMC Cancer. 2005 Jul 7;5:75. doi: 10.1186/1471-2407-5-75 (PMC1182358; doi:10.1186/1471-2407-5-75)
Supplement: Additional File 1 — Summary of the top 10 differentially expressed genes in Caco-2, at all time points studied. [file 1471-2407-5-75-S1.doc]

| **Regulation** | **Time** | **Affymetrix Probe Set ID** | **Gene** | **Fold change** | **Genbank** | **Description** |
| --- | --- | --- | --- | --- | --- | --- |
| UP | 1h | 1978_at | ITGA2 | 1,50 | X17033 | integrin, alpha 2 |
|  |  |  |  |  |  |  |
|  | 12h | 1025_g_at | CYP1A1 | 5,25 | X02612 | Human gene for cytochrome P(1)-450. |
|  |  | 36767_at | CYP1A1 | 4,80 | K03191 | cytochrome P450 |
|  |  | 39248_at | AQP3 | 3,33 | N74607 | aquaporin 3 |
|  |  | 32193_at | PLXNC1 | 3,12 | AF030339 | plexin C1 |
|  |  | 34363_at | SEPP1 | 2,60 | Z11793 | selenoprotein P, plasma, 1 |
|  |  | 1024_at | CYP1A1 | 2,52 | X02612 | Human gene for cytochrome P(1)-450. |
|  |  | 37399_at | AKR1C3 | 2,33 | D17793 | aldo-keto reductase family 1, member C3 |
|  |  | 39809_at | HBP1 | 2,31 | AF019214 | HMG-box transcription factor 1 |
|  |  | 36933_at | NDRG1 | 2,23 | D87953 | N-myc downstream regulated gene 1 |
|  |  | 32787_at | ERBB3 | 2,22 | M34309 | v-erb-b2 erythroblastic leukemia viral oncogene homolog 3 (avian) |
|  |  |  |  |  |  |  |
|  | 24h | 36767_at | CYP1A1 | 3,87 | K03191 | cytochrome P450 |
|  |  | 39248_at | AQP3 | 3,12 | N74607 | aquaporin 3 |
|  |  | 37399_at | AKR1C3 | 2,53 | D17793 | aldo-keto reductase family 1 |
|  |  | 35345_at | HMGCS2 | 2,37 | X83618 | 3-hydroxy-3-methylglutaryl-Coenzyme A synthase 2 (mitochondrial) |
|  |  | 32193_at | PLXNC1 | 2,22 | AF030339 | plexin C1 |
|  |  | 614_at | PLA2G2A | 2,15 | M22430 | phospholipase A2, group IIA (platelets, synovial fluid) |
|  |  | 38717_at | DKFZP586A0522 | 1,98 | AL050159 | DKFZP586A0522 protein |
|  |  | 32664_at | RNASE4 | 1,96 | D37931 | ribonuclease, RNase A family, 4 |
|  |  | 37684_at | SLC21A9 | 1,94 | AB020687 | solute carrier organic anion transporter family, member 2B1 |
|  |  | 38582_at | SPINK1 | 1,92 | AI961220 | serine protease inhibitor, Kazal type 1 |
|  |  |  |  |  |  |  |
| DOWN | 1h | 41193_at | DUSP6 | -2,72 | AB013382 | dual specificity phosphatase 6 |
|  |  | 33389_at | CYP51A1 | -1,68 | U23942 | cytochrome P450, family 51, subfamily A, polypeptide 1 |
|  |  | 41446_f_at | MT1F | -1,58 | H68340 | metallothionein 1F (functional) |
|  |  | 1916_s_at | FOS | -1,58 | V01512 | Human cellular oncogene c-fos (complete sequence). |
|  |  | 36618_g_at | ID1 | -1,57 | X77956 | inhibitor of DNA binding 1 |
|  |  | 34840_at |  | -1,54 | AI700633 | Homo sapiens cDNA: FLJ22642 fis, clone HSI06970 |
|  |  | 39248_at | AQP3 | -1,52 | N74607 | aquaporin 3 |
|  |  | 37141_at | FOXA1 | -1,49 | U39840 | forkhead box A1 |
|  |  |  |  |  |  |  |
|  | 12h | 38772_at | CYR61 | -3,59 | Y11307 | cysteine-rich, angiogenic inducer, 61 |
|  |  | 39215_at | NPPB | -2,42 | AL021155 | Human DNA sequence from clone RP5-934G17 |
|  |  | 38422_s_at | FHL2 | -2,31 | U29332 | four and a half LIM domains 2 |
|  |  | 31888_s_at | TSSC3 | -2,31 | AF001294 | pleckstrin homology-like domain, family A, member 2 |
|  |  | 36790_at | TPM1 | -1,88 | M19267 | tropomyosin; Human tropomyosin mRNA, complete cds. |
|  |  | 40114_at |  | -1,86 | J00077 | Human mRNA encoding alpha-fetoprotein (AFP) |
|  |  | 38700_at | CSRP1 | -1,85 | M33146 | cysteine and glycine-rich protein 1 |
|  |  | 35803_at | ARHE | -1,84 | S82240 | ras homolog gene family, member E |
|  |  | 40079_at |  | -1,84 | AA156240 | retinoic acid induced 3 |
|  |  | 33328_at |  | -1,84 | W28612 | 49b3 Human retina |
|  |  |  |  |  |  |  |
|  | 24h | 38772_at | CYR61 | -4,36 | Y11307 | cysteine-rich, angiogenic inducer, 61 |
|  |  | 39215_at | NPPB | -3,12 | AL021155 | Human DNA sequence from clone RP5-934G17 |
|  |  | 33328_at |  | -2,52 | W28612 | 49b3 Human retina |
|  |  | 38842_at | AMOTL2 | -2,40 | AB023206 | angiomotin like 2 |
|  |  | 40855_at | KIAA1053 | -2,32 | AB028976 | sterile alpha motif domain containing 4 |
|  |  | 38422_s_at | FHL2 | -2,17 | U29332 | four and a half LIM domains 2 |
|  |  | 37679_at | IFRD1 | -2,07 | Y10313 | interferon-related developmental regulator 1 |
|  |  | 32901_s_at | WUGSC | -2,05 | AC005192 | similar to mouse interferon-related protein PC4 |
|  |  | 40374_at | CARP | -2,05 | X83703 | ankyrin repeat domain 1 (cardiac muscle) |
|  |  | 39695_at | DAF | -2,04 | M31516 | decay accelerating factor for complement |
