# Supplementary material for: Global gene expression analysis in time series following N-acetyl L-cysteine induced epithelial differentiation of human normal and cancer cells in vitro
Source: BMC Cancer. 2005 Jul 7;5:75. doi: 10.1186/1471-2407-5-75 (PMC1182358; doi:10.1186/1471-2407-5-75)
Supplement: Additional File 2 — Summary of the top 10 differentially expressed genes in NHEK, at all time points studied. [file 1471-2407-5-75-S2.doc]

| **Regulation** | **Time** | **Affymetrix Probe Set ID** | **Gene** | **Fold change** | **Genbank** | **Description** |
| --- | --- | --- | --- | --- | --- | --- |
| UP | 1h | 287_at | ATF3 | 2,60 | L19871 | activating transcription factor 3 |
|  |  | 34703_f_at |  | 2,22 | AA151971 | Homo sapiens cDNA clone IMAGE:588365 |
|  |  | 34702_f_at | HUMRTVLH3 | 2,11 | M27826 | Homo sapiens human endogeneous retrovirus RTVL-H neutral protease |
|  |  | 1011_s_at | YWHAE | 1,97 | U54778 | tyrosine 3-monooxygenase |
|  |  | 35798_at | VPS28 | 1,85 | W25936 | glutamate receptor |
|  |  | 1764_s_at | MAZ | 1,82 | D85131 | MYC-associated zinc finger protein |
|  |  | 40357_at | INHBA | 1,72 | J03634 | inhibin, beta A |
|  |  | 37028_at | PPP1R15A | 1,70 | U83981 | protein phosphatase 1 |
|  |  | 40489_at | DRPLA | 1,69 | D31840 | atrophin-1 |
|  |  | 41565_at | A2LP | 1,67 | AF034373 | Homo sapiens ataxin-2-like protein A2LP (A2LG) mRNA |
|  |  |  |  |  |  |  |
|  | 12h | 31859_at | MMP9 | 3,59 | J05070 | matrix metalloproteinase 9 |
|  |  | 38404_at | TGM2 | 2,43 | M55153 | transglutaminase 2 |
|  |  | 1715_at | TNFSF10 | 2,35 | U37518 | tumor necrosis factor (ligand) superfamily, member 10 |
|  |  | 40357_at | INHBA | 2,11 | J03634 | inhibin, beta A |
|  |  | 1385_at | TGFBI | 1,93 | M77349 | transforming growth factor |
|  |  | 31508_at | TXNIP | 1,87 | S73591 | thioredoxin interacting protein |
|  |  | 39809_at | HBP1 | 1,82 | AF019214 | HMG-box transcription factor 1 |
|  |  | 36980_at | PROL2 | 1,81 | U03105 | proline-rich nuclear receptor coactivator 1 |
|  |  | 36117_at | PTK2 | 1,81 | L13616 | PTK2 protein tyrosine kinase 2 |
|  |  | 312_s_at |  | 1,80 |  |  |
|  |  |  |  |  |  |  |
|  | 24h | 31859_at | MMP9 | 5,79 | J05070 | matrix metalloproteinase 9 |
|  |  | 38404_at | TGM2 | 4,50 | M55153 | transglutaminase 2 |
|  |  | 40357_at | INHBA | 3,04 | J03634 | inhibin, beta A |
|  |  | 33439_at | TCF8 | 2,90 | D15050 | 7 zinc finger domains, DNA binding protein homeodomain |
|  |  | 32242_at | CRYAB | 2,54 | AL038340 |  |
|  |  | 35959_at | KIAA0844 | 2,53 | AB020651 | zinc finger protein 365 |
|  |  | 34517_at | HMGCS1 | 2,51 | X66435 | 3-hydroxy-3-methylglutaryl-Coenzyme A synthase 1 |
|  |  | 36502_at | PFTK1 | 2,44 | AB020641 | PFTAIRE protein kinase 1 |
|  |  | 32243_g_at | CRYAB | 2,43 | AL038340 |  |
|  |  | 40717_at | CTSL2 | 2,41 | AB001928 | cathepsin L2 |
|  |  |  |  |  |  |  |
| DOWN | 1h | 34517_at | HMGCS1 | -2,00 | X66435 | 3-hydroxy-3-methylglutaryl-Coenzyme A synthase 1 (soluble) |
|  |  | 32706_at | HIRA | -1,96 | X89887 | HIR histone cell cycle regulation defective homolog A (S. cerevisiae) |
|  |  | 39827_at | RTP801 | -1,94 | AA522530 | HIF-1 responsive RTP801 |
|  |  | 434_at | H1F0 | -1,86 | X03473 | unnamed protein product; histone H1(0) |
|  |  | 36498_at | AP1S1 | -1,73 | AI936759 | Homo sapiens transcribed sequences |
|  |  | 1343_s_at | SERPINB3 | -1,66 | S66896 | serine (or cysteine) proteinase inhibitor, clade B (ovalbumin), member 3 |
|  |  | 38288_at | SNAI2 | -1,65 | U69196 | snail homolog 2 (Drosophila) |
|  |  | 41800_s_at | DNAJC7 | -1,64 | U46571 | DnaJ (Hsp40) homolog, subfamily C, member 7 |
|  |  | 35963_at | HKE2 | -1,64 | AI201243 | HLA class II region expressed gene KE2 |
|  |  | 41471_at | S100A9 | -1,64 | W72424 | S100 calcium binding protein A9 (calgranulin B) |
|  |  |  |  |  |  |  |
|  | 12h | 408_at | CXCL1 | -2,27 | X54489 | Human gene for melanoma growth stimulatory activity (MGSA). |
|  |  | 1148_s_at |  | -1,96 |  |  |
|  |  | 658_at | THBS2 | -1,85 | L12350 | thrombospondin 2 |
|  |  | 32719_at | NRG1 | -1,85 | L41827 | neuregulin 1 |
|  |  | 1536_at | CDC6 | -1,61 | U77949 | CDC6 cell division cycle 6 homolog (S. cerevisiae) |
|  |  | 1516_g_at |  | -1,58 |  |  |
|  |  | 659_g_at | THBS2 | -1,57 | L12350 | thrombospondin 2 |
|  |  | 32215_i_at | RHOBTB3 | -1,56 | AB020685 | Rho-related BTB domain containing 3 |
|  |  | 37324_at | TFRC | -1,54 | X01060 | transferrin receptor (p90, CD71) |
|  |  | AFFX-HUMTFRR/M11507_5_at | TFRC | -1,51 | M11507 | transferrin receptor (p90, CD71) |
|  |  |  |  |  |  |  |
|  | 24h | 1148_s_at |  | -2,67 |  |  |
|  |  | 408_at | CXCL1 | -2,55 | X54489 | Human gene for melanoma growth stimulatory activity (MGSA). |
|  |  | 1945_at | CCNB1 | -2,55 | M25753 | cyclin B1 |
|  |  | 38414_at | CDC20 | -2,40 | U05340 | CDC20 cell division cycle 20 homolog (S. cerevisiae) |
|  |  | 1943_at | CCNA2 | -2,39 | X51688 | cyclin A2 |
|  |  | 658_at | THBS2 | -2,34 | L12350 | thrombospondin 2 |
|  |  | 893_at | E2-EPF | -2,34 | M91670 | ubiquitin carrier protein |
|  |  | 34852_g_at | STK6 | -2,33 | AF011468 | serine/threonine kinase 6 |
|  |  | 36837_at | KIF2C | -2,30 | U63743 | kinesin family member 2C |
|  |  | 1721_g_at | MAD2L1 | -2,27 | U65410 | MAD2 mitotic arrest deficient-like 1 (yeast) |
